# Size and Zeta Potential Clicked Germination Attenuation and Anti-Sporangiospores Activity of PEI-Functionalized Silver Nanoparticles against COVID-19 Associated Mucorales (*Rhizopus arrhizus*)

**DOI:** 10.3390/nano12132235

**Published:** 2022-06-29

**Authors:** Atul Kumar Tiwari, Munesh Kumar Gupta, Govind Pandey, Ragini Tilak, Roger J. Narayan, Prem C. Pandey

**Affiliations:** 1 Department of Chemistry, Indian Institute of Technology (BHU), Varanasi 221005, India; atulkumartiwari.rs.chy19@itbhu.ac.in; 2 Mycology Laboratory, Department of Microbiology, Institute of Medical Sciences, Banaras Hindu University, Varanasi 221005, India; muneshg.micro@bhu.ac.in (M.K.G.); tilakragini28@gmail.com (R.T.); 3 Department of Paediatrics, King George Medical University, Lucknow 226003, India; gvnd121@gmail.com; 4 Joint Department of Biomedical Engineering, North Carolina State University, Raleigh, NC 27695, USA

**Keywords:** germination, COVID-19 associated mucormycosis, SERS, CLSM, SARS-CoV-2, black fungus

## Abstract

The SARS-CoV-2 infections in Indian people have been associated with a mucormycotic fungal infection caused by the filamentous fungi *Rhizopus arrhizus*. The sporangiospores of *R. arrhizus* are omnipresent in the environment and cause infection through inhalation or ingestion of contaminated air and foods. Therefore, the anti-sporangiospore activity of polyethyleneimine functionalized silver nanoparticles (PEI-f-Ag-NPs) with variable size and surface charge as a function of the molecular weight of PEI was explored. The results showed that both PEI-f-AgNP-1 and PEI-f-AgNP-2, potentially, attenuated the germination and reduced the viability of sporangiospores. Furthermore, the results showed that the minimum inhibitory concentration (MIC) values of both PEI-f-AgNP-1 and PEI-f-AgNP-2 (1.65 and 6.50 μg/mL, respectively) were dependent on the nanoparticle size and surface ζ potentials. Similarly, the sporangiospore germination inhibition at MIC values was recorded, showing 97.33% and 94% germination inhibition, respectively, by PEI-f-AgNP-1 and 2 within 24 h, respectively. The confocal laser scanning microscopy, SEM-EDS, and confocal Raman spectroscopy investigation of PEI-f-Ag-NPs treated sporangiospores confirmed size and surface charge-dependent killing dynamics in sporangiospores. To the best of our knowledge, this is the first investigation of the polyethyleneimine functionalized silver nanoparticle-mediated size and surface charge-dependent anti-sporangiospore activity against *R. arrhizus,* along with a possible antifungal mechanism.

## 1. Introduction

Mucormycosis is an angio-invasive fungal infection that is caused by saprophytic fungi belonging to the order *Mucorales*. Infection is associated with complications that are characterized by tissue necrosis and infarction of blood vessels [1,2,3]. A comparison of global data on mucormycosis indicates that the case burden of mucormycosis was estimated to be 70 times higher in India during the COVID-19 pandemic [4,5]. A multi-country study on COVID-19-associated mucormycosis (CAM) reported that 53% of the cases were from India, followed by the United States of America (10%), Pakistan (6.3%), France (5%), Mexico (5%), Iran (5%), and Russia (2.5%) [6]. During the second wave associated with the COVID-19 pandemic, the caseload of mucormycosis increased drastically (>50,000 cases) in India, prompting the Indian health authorities to declare mucormycosis as an endemic disease [7]. The situation is challenging due to the limited availability of first-line antifungal drugs, such as liposomal amphotericin and amphotericin B deoxycholate. Because of the high abundance of clinically relevant *Mucorales* in Indian soils, sporangiospores are highly prevalent in indoor and outdoor air [2,8]. Patients acquire the infection by inhalation, ingestion, or traumatic inoculation of the viable sporangiospores from the environment. Morphologically, the *Mucorales* sporangiospores (especially *R. arrhizus* sporangiospores) are ~2–5 μm in size; the sporangiospore wall exhibits a rough, amorphous outer surface and a fibrillar inner surface. Structurally, the spore wall can be composed of three layers: (a) an outer characteristic electron-opaque layer with a thickness of 10–240 nm, sometimes covered by a fragile extra layer, (b) an electron-dense ~160–245 nm thick layer, and (c) an innermost layer of relatively high electron density covering the plasma membrane (~15–40 nm thick). Chemically, the major spore wall components are proteins, glucan, chitosan, and melanin, followed by smaller amounts of uronic acids, lipids, chitin, and mannose. The protein(s) contain high aspartic acid and glutamic acid levels, followed by glycine, alanine, lysine, histidine, and serine, and smaller amounts of other amino acids.

The antifungal activity of silver nanoparticles is well documented in various fungal species; however, the metal nanoparticle-mediated sporicidal activity of *Mucorales* member *R. arrhizus* is underreported to this point. Silver cations are considered a Lewis acid, which reacts with a Lewis base; Lewis bases include phosphorous and sulfur-containing biomolecules, which are found in significant components of the cell wall, cell membrane, proteins, and DNA bases [9]. Mechanistically, silver nanoparticles can cover the cell surface through non-specific interaction with biomolecules on the cell wall and membrane, leading to morphological changes, such as shrinkage of the cytoplasm, membrane detachment, and the formation of numerous electron-dense pits, which ultimately cause cell death [10,11,12]. A hydrophilic, cationic polymer coating on silver nanoparticles may allow for the selective interaction of silver nanoparticles with the microbial cell; as such, enhanced antifungal activity can be observed. Incorporating a cationic charge on nanoparticles and other types of inorganic materials entails surface modification with cationic polymers (e.g., poly-amidoamine, poly-ethyleneimine (PEI), and poly-lysine) via covalent or electrostatic mechanisms and surface grafting with amine groups [13,14,15]. In addition, in the process of nanoparticle formation, the size may further be controlled as a function of molecular weight (*Mw*) and the working concentration of the polymer. In our present study, we have assessed the germination inhibition and anti-sporangiospores activity of PEI-functionalized Ag-NPs as a function of PEI molecular weight (*Mw* 700,000 Da and 65,000 Da), which modulated surface charge and size of silver nanoparticles, against clinically isolated *R. arrhizus*.

## 2. Materials and Methods

### 2.1. Materials

The polyethyleneimine (PEI), silver nitrate, and propidium iodide (PI) were obtained from Sigma-Aldrich (Bangalore, Karnataka, India). Fungal culture media, such as RPMI (Roswell Park Memorial Institute, Buffalo, NY, USA), Sabouraud dextrose agar (SDA), and broth, were collected from Hi-Media Laboratories Ltd. (Mumbai, Maharashtra, India). Plasticware was purchased from Tarsons Products Private Ltd. (Kolkata, West Bengal, India). Amphotericin B and other routine chemicals were purchased from Sigma-Aldrich (St. Louis, MO, USA). Finally, solvents were purchased from Merck Life Science Private Limited (Bangalore, Karnataka, India). All the reagents were of analytical grade.

### 2.2. Fungal Strain

This study was approved by the Institution Ethical Committee, Institute of Medical Sciences, at Banaras Hindu University, Varanasi (Dean/2021/EC/3003, dated 29 October 2021). Here, we used a clinical strain of *R. arrhizus* isolated from a 46-years-old male suffering from rhino-orbital mucormycosis during the second wave of the SARS-CoV2 infection in India. The strain was identified as per standard mycological procedure, including culture and morphological characteristics. For further experiments, the isolated strain was sub-cultured in SD broth and preserved in 25% glycerol at −80 °C.

### 2.3. Harvesting of Sporangiospores

The preserved culture was sub-cultured on a Sabouraud dextrose agar slant for 4–6 days at 30 °C, and the sporangiospores of *R. arrhizus* were harvested as per the method of Singh et al. 2011. [16] In brief, a total of 10 mL of 0.9% saline solution was then added to the slant tube, followed by repeated washing of the mycelia with the added saline. The resulting suspension was collected and centrifuged at 3500× *g* for 8 min; the pellet was washed with 0.1 M PBS and resuspended in 0.9% saline. The spores were counted using a Neubauer hemacytometer, and the counts were expressed as sporangiospores/mL.

### 2.4. Synthesis and Physical Characterization of PEI Functionalized Silver Nanoparticles

The PEI functionalized silver nanoparticles (PEI-f-AgNP-1 and 2) were synthesized as described previously by Tiwari et al. 2020 with a slight modification [17]. In short, 120 µL of ethylene glycol and a methanolic 1-vinyl 2-pyrrolidone solution (50 µL of a 250 mM solution) were placed in 2 mL glass vial, followed by the inclusion of a methanolic AgNO_3_ solution (20 µL of a 10 mM solution), PEI 1 (*Mw* 700,000) and 2 (*Mw* 65,000) (50 and 25 µL of a 20 mg/mL solution, respectively, in separate vials), and cyclohexanone (20 μL). The reaction mixture was thoroughly mixed using a vortex mixer for 30 s and then placed in a microwave oven for 10 s. The cycle was repeated 4–6 times in a microwave oven, leading to the formation of a deep yellow color that signified the formation of PEI-f-Ag-NP-1 and 2. The synthesized silver nanoparticles were dispersible in water without any physical changes. All the experiments were conducted with a dilution of silver nanoparticles in ultrapure water.

All the polyethyleneimine functionalized silver nanoparticles (PEI-f-Ag-NPs) were characterized using a U-2900 UV-Vis spectrophotometer (Hitachi, Tokyo, Japan) over the scan range of 250–800 nm. Selected area electron diffraction and transmission electron microscopy analysis of Ag-NPs were carried out using a Tecnai G20 Twin instrument (FEI, Hillsboro, OR, USA). Samples were prepared by diluting the Ag-NPs in methanol and drop-casting the solution on carbon-coated copper grids of 300 mesh. Zeta potential analysis was performed using a Zetasizer instrument (Malvern Panalytical, Malvern, UK). All graphs were plotted on Origin 2018 software (OriginLab Corporation, Northampton, MA, USA).

### 2.5. Assessment of In Vitro Antifungal Activity of Silver Nanoparticles

The 4–6 day grown culture on SD agar of isolated *R. arrhizus* was prepared and the sporangiospores were harvested, as described previously. The harvested sporangiospores were washed with PBS twice, resuspended in RPMI liquid medium, and adjusted to 2 × 10^7^ spores per mL in a 5 mL test suspension. 50 µg (100 µL of stock solution) of synthesized silver nanoparticles (PEI-f-AgNP-1 and 2) were inoculated in each test suspension. Similarly, amphotericin B (2 µg/mL) was taken as the positive control, and a distilled water inoculated suspension was taken as the negative control. All the test samples were incubated at 28 °C for 72 h. To assess the sporangiospore morphology after treatment with PEI-f-Ag-NP-1 and 2, a 10 µL of treated aliquot was obtained at various time intervals (24, 48, and 72 h) from each tube, placed on a glass slide, covered with glass slip, and observed under a bright-field binocular compound light microscope (Olympus, Tokyo, Japan) with 40× objective lenses. The anti-sporangiospore activity was confirmed by inoculating 5 µL of treated aliquots on SD agar plates. All the tests were repeated in triplicate, and average values were determined. The spore diameter of the ungerminated (freshly harvested), germinated (negative control), and PEI-f-Ag-NPs treated samples was measured using the following formula:

Cell diameter (per pixel) = Physical length of a pixel on the CCD/total magnification

The spore diameter was calculated with the help of ImageJ software (NIH, Bethesda, MD, USA), and the values were plotted using Origin 8.5 2018 software (OriginLab Corporation, Northampton, MA, USA) to create statistical histograms.

### 2.6. Minimum Inhibitory Concentration (MIC) Determination of PEI-f-Ag-NPs

The MIC data for PEI-functionalized silver nanoparticles (PEI-f-AgNP-1 and 2) against *R. arrhizus* sporangiospores (0.5–5 × 10^4^ spores/mL) were obtained using the two-fold serial dilution method in a flat-bottom sterile 96-well microtiter plate. An active suspension of 50 µg/mL PEI-f-Ag-NPs was prepared; 100 µL of each suspension was distributed in the wells using the double dilution approach; the final concentration was between 0.10 and 50 µg/mL. Subsequently, 100 µL of the sporangiospore suspension was added to each well. Amphotericin B and sterile distilled water were used as a positive control and negative control, respectively. The microtiter plate was incubated in a static position at 28 °C for 72 h; visual demonstration of turbidity (i.e., a visually clear well) was recorded as the MIC. After that, a 10 µL aliquot from each well was sub-cultured on the SD agar plates for 72 h; fungal growth was subsequently observed. The MIC data were determined as the concentration of PEI-f-Ag-NPs at which the growth was inhibited or slowed compared to the standard control.

### 2.7. Studies on Germination Attenuation of Sporangiospores

*R. arrhizus* sporangiospores were prepared, as described previously [16]. The spore germination inhibition assay was performed, as described previously [18]. Briefly, 100 µL of sporangiospore suspensions (10^7^ spores/mL) were mixed with PEI-f-Ag-NP 1 and 2 in the separate tubes with a final concentration of 0.41, 0.82, 1.65, and 3.30 μg/mL for PEI-f-AgNP-1 and 1.62, 3.25, 6.50, and 13.0 µg/mL for PEI-f-AgNP-2. Control samples (positive and negative) contained sporangiospores suspensions with 50 µL (0.50 µg/mL) of amphotericin B and 50 µL of distilled water, respectively. The 50 µL mixture with a different concentration of PEI-f-Ag-NPs was transferred onto a sterile concave slide for incubation at 28 °C. Three slides were prepared for each treatment, and mean values were compared. A hundred sporangiospores per treatment were observed at an interval of 24 h for germination inhibition of sporangiospores using a bright-field binocular compound light microscope (Olympus, Tokyo, Japan). The spore germination rate was calculated as follows [18]:

Spore germination rate (%) = (the number of germinated spores)/(total number of spores)

### 2.8. Cell Morphology Observation with Scanning Electron Microscopy (SEM)

The ultrastructural changes in PEI-f-AgNP-1 and 2 treated sporangiospores were investigated using SEM. Initially, the sporangiospore suspensions were treated with PEI-f-Ag-NP-1 and 2 for 24 h at 28 °C, followed by centrifugation at 3500 rpm for 6 min. The condensed cells were fixed with 2.5% glutaraldehyde and postfixed with 1% aqueous OsO_4_, followed by washing with 0.1 M (pH 7.0) phosphate buffers. Subsequently, the samples were dehydrated in an ascending concentration of ethanol in a serial manner at 30, 50, 70, 80, 90, and 100% for 15 min and dried in a vacuum oven. Finally, the cells were placed on the glass coverslip, coated with gold, and observed using a JSM-6700F SEM (JEOL, Tokyo, Japan).

### 2.9. Confocal Laser Scanning Microscopy (CLSM) Studies

Confocal microscopic examination was performed to assess the viability of sporangiospores incubated with PEI-f-Ag-NP-1 and 2 [18]. Briefly, 5 mL of freshly prepared sporangiospore suspensions (2 × 10^7^ spores/mL) was prepared as described above, and treated with 50 µL of PEI-f-Ag-NP 1 and 2 each at its MIC value in a separate tube along with positive and negative controls (amphotericin B and distilled water) at 28 °C for 72 h. A 1 mL of aliquot was withdrawn every 24 h interval from each tube and centrifuged at 3500 rpm for 5 min. The pellet was washed twice with PBS (0.1 M) and resuspended in PBS. The spores were stained with 10 µL (1 mg/mL stock) of propidium iodide (PI) (excitation/emission at 535 nm/617 nm) for 25 min in the dark and washed again to eliminate excess dye with PBS (phosphate buffer saline). Subsequently, the treated samples were observed under an SP5 AOBS confocal lesser scanning microscope (Leica, Wetzlar, Germany) with a 40× immersion oil objective. The anti-sporangiospore activity of PEI-f-Ag-NP-1 and 2 were confirmed by the fact that dead (damaged cell membrane) sporangiospores stain with propidium iodide and live spores remained unstained since intact plasma membrane prevents the passage of potassium iodide (PI) inside the cell.

### 2.10. Surface Enhanced Raman Spectroscopy (SERS) Studies

For the confocal Raman spectroscopic study, the PEI-f-Ag-NP-1 and 2 treated and untreated sporangiospores suspensions were centrifuged at 3500 rpm for 6 min. The semi-solid suspension was spotted on a cover glass and dried under ambient conditions. Confocal Raman measurements were obtained using a CRM Alpha 300 S instrument (WiTec GmbH, Ulm, Germany). The excitation source for this study was a 532 nm Nd: YAG laser, and the maximum power output of the laser was 40 mW. The laser power was attenuated to ≈10 mW to the sampling point. Measurements were performed using a 100× objective. The dispersed light intensity of the signal from the grating was measured by a Peltier-cooled charge-coupled device (CCD). The maximum area of laser exposure was 750 nm, and the collection was performed confocally. Spectra were collected in the 400–2100 cm^−1^ window [19]. The band peaks were matched with respective wavenumber values from the work of Joke De Gelder et al. [20].

## 3. Results

### 3.1. Synthesis and Characterization of PEI Functionalized Silver Nanoparticles

**Figure 1:**
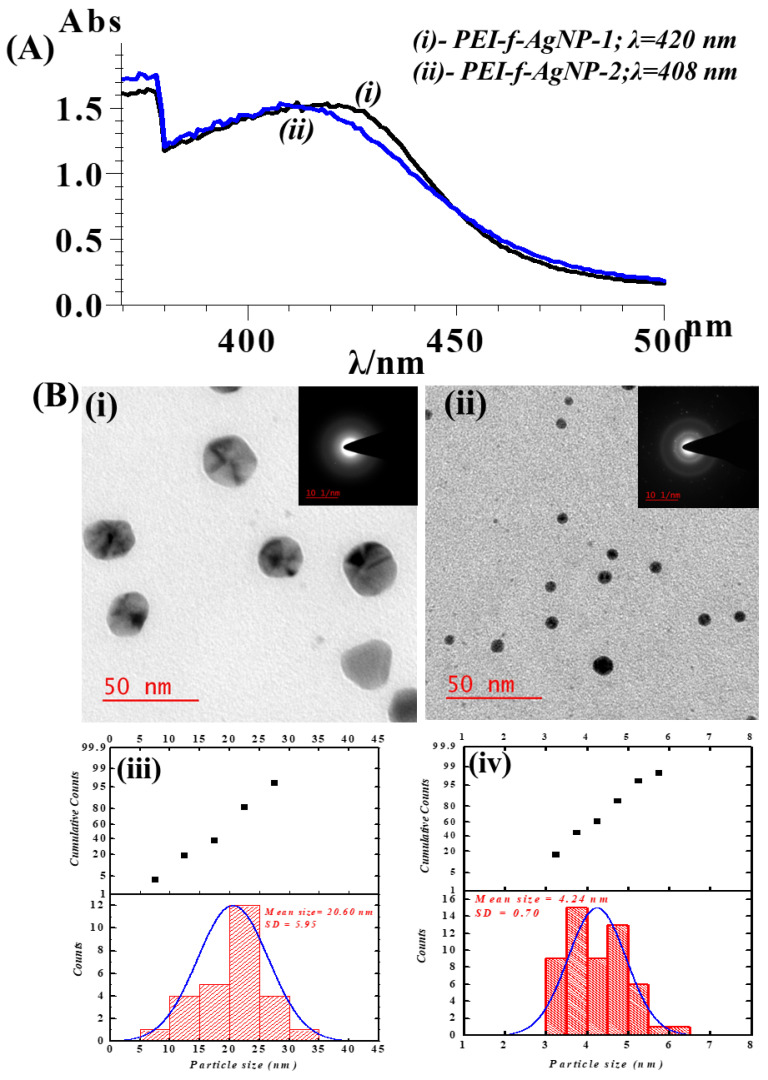
Physical characterization of PEI-f-Ag-NPs. (**A**) UV-Vis spectra of PEI-f-AgNP-1 (**i**) showing an absorption maximum at λ = 420 nm and PEI-f-AgNP-2 (**ii**) showing an absorption maximum at showing at λ = 408 nm. (**B**) shows the TEM image and size histogram of PEI-f-AgNP-1 (**i**,**iii**) as well as the TEM image and size histogram of PEI-f-AgNP-2 (**ii**,**iv**).

**Figure 2:**
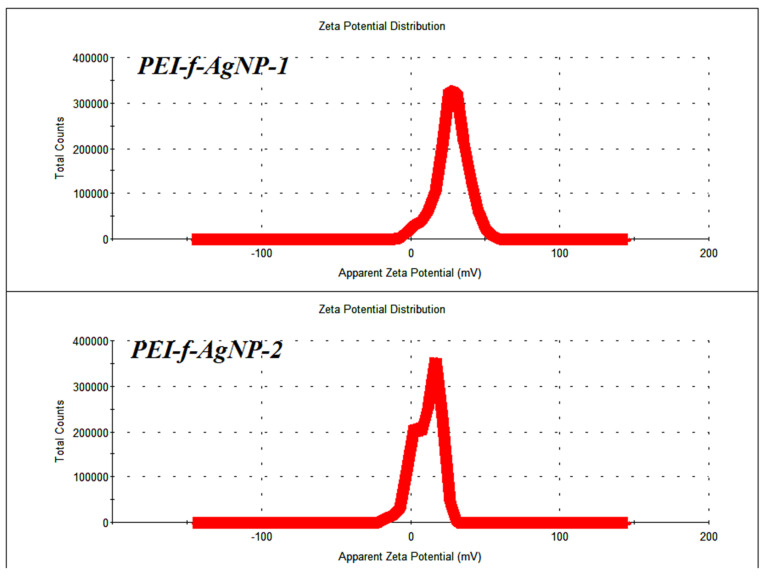
Zeta potential distribution of PEI-f-AgNP-1 and 2.

Polyethyleneimine (PEI) mediated synthesis of gold nanoparticles under ambient conditions was reported previously by Pandey et al. [21,22]. The variation of the molecular weight of PEI functionalized silver cations (PEI-f-Ag^+^) under microwave-assisted synthesis and in the presence of 1-vinyl 2-pyrrolidone as a stabilizer and organic reducing agent, cyclohexanone, led to the rapid and size-controlled synthesis of silver nanoparticles [18]. Figure 1A(i,ii) shows the UV-Vis spectrum of synthesized silver nanoparticles functionalized with PEI of two different *Mw*s; PEI-f-AgNP-1 was synthesized with 700,000 Da PEI and PEI-f-AgNP-2 was synthesized with 65,000 Da PEI. Figure 1A(i) shows the results recorded for PEI-f-Ag-NP-1 (λ = 420 nm) and Figure 1A(ii) shows the results recorded for PEI-f-Ag-NP-2 (λ = 408 nm). This optimized process allowed for the rapid formation of PEI-f-Ag-NPs, regardless of the molecular weight of PEI. The physical properties of PEI-f-Ag-NP-1 and 2 differed, based on the molecular weight of PEI. The shift in the UV-Vis spectrum indicated that the higher molecular weight PEI (PEI-f-AgNP-1, mean size 20.6 nm) was associated with larger-sized silver nanoparticles than the lower molecular weight PEI (PEI-f-Ag-NP-2, mean size 4.2 nm). The UV-Vis results were also supported by transmission electron microscopy (TEM) analysis of the PEI-f-Ag-NPs, as shown in Figure 1B(i–iv). The recorded zeta potential of the synthesized Ag-NPs indicated that the molecular weight of PEI controls not only the size of the silver nanoparticles but also the surface charge of the nanoparticles. Figure 2 shows the recorded zeta potential of PEI-f-AgNP-1 and 2 of 25 ± 2 and 16 ± 1.5 mV, respectively (Figure 2). It was noted the PEI-f-Ag-NPs surface charges were depended on the molecular weight of polyethyleneimine. The PEI-f-Ag-NP-1 was associated with a higher surface charge (25 ± 2 mV) and larger nanoparticle size (~20.4 nm) than PEI-f-AgNP-2.

### 3.2. In Vitro Anti-Sporangiospore Activity, Particularly the Effect of PEI-f-Ag-NPs on Sporangiospore Size and Minimum Inhibitory Concentration Value

**Figure 3:**
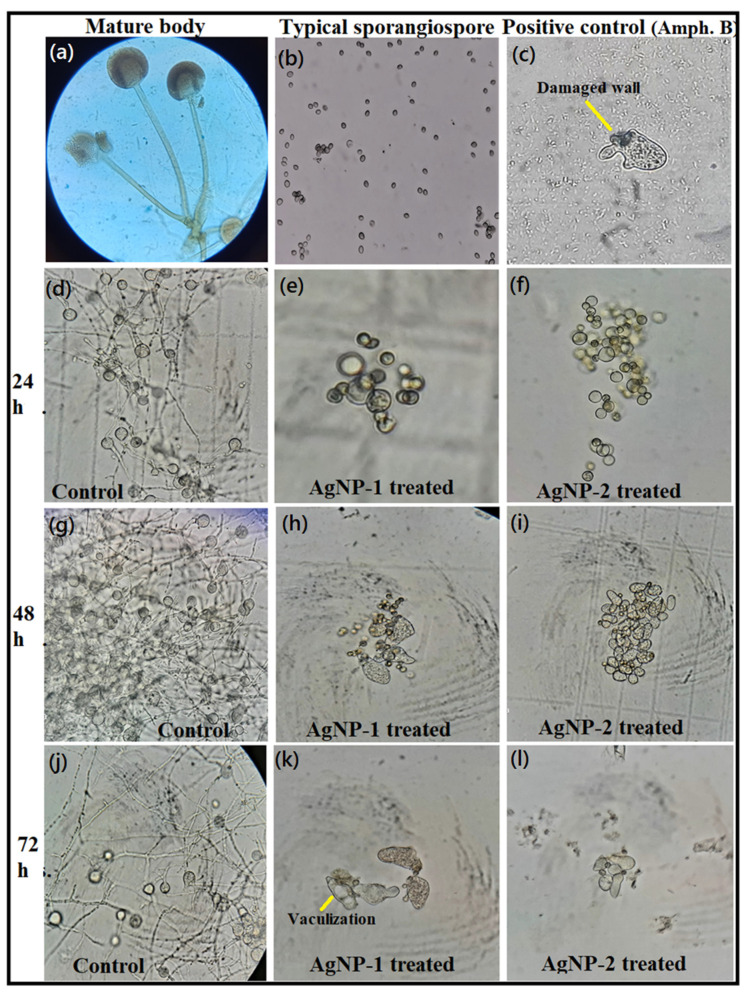
Assessment of anti-sporangiospore activity of PEI-f-Ag-NPs. (**a**) Wet mounted vegetative body of *R. arrhizus* with sporangiospore, (**b**) freshly harvested sporangiospores, (**c**) sporangiospores treated with amphotericin B for 24 h, (**d**) control sporangiospores at 24 h, (**e**) treated with PEI-f-AgNP-1 for 24 h, (**f**) treated with PEI-f-AgNP-2 for 24 h, (**g**) control sporangiospores at 48 h, (**h**) treated with PEI-f-AgNP-1 for 48 h, (**i**) treated with PEI-f-AgNP-2 for 48 h, (**j**) control sporangiospores at 72 h, (**k**) treated with PEI-f-AgNP-1 for 72 h, and (**l**) treated with PEI-f-AgNP-2 for 72 h.

The germination of dormant spores is the first crucial step in returning the life cycle of spores to vegetative growth [18]. In this study, we assessed the anti-sporangiospore activity of synthesized PEI-f-AgNPs-1 and 2, which differed in terms of size and surface charge. The assessment was conducted for 72 h. Every 24 h, an aliquot was taken; morphological changes were captured using a bright-field compound light microscope along with measurements of sporangiospore size. It was observed that PEI-f-AgNP-1 and 2 have a varied effect on sporangiospore characteristics. Initially, after 24 h of incubation with PEI-f-AgNP-1, some surface deformation was observed. However, PEI-f-AgNP-2 treated sporangiospores did not show significant changes except germination attenuation. The observed morphological changes are shown in Figure 3. At later time points (e.g., 48 and 72 h), both PEI-f-Ag-NPs treated sporangiospores showed surface deformations and vacuolization in a few populations. These findings indicate differences in killing dynamics based on the surface charge (ζ potential) and size of PEI-f-Ag-NPs.

**Figure 4:**
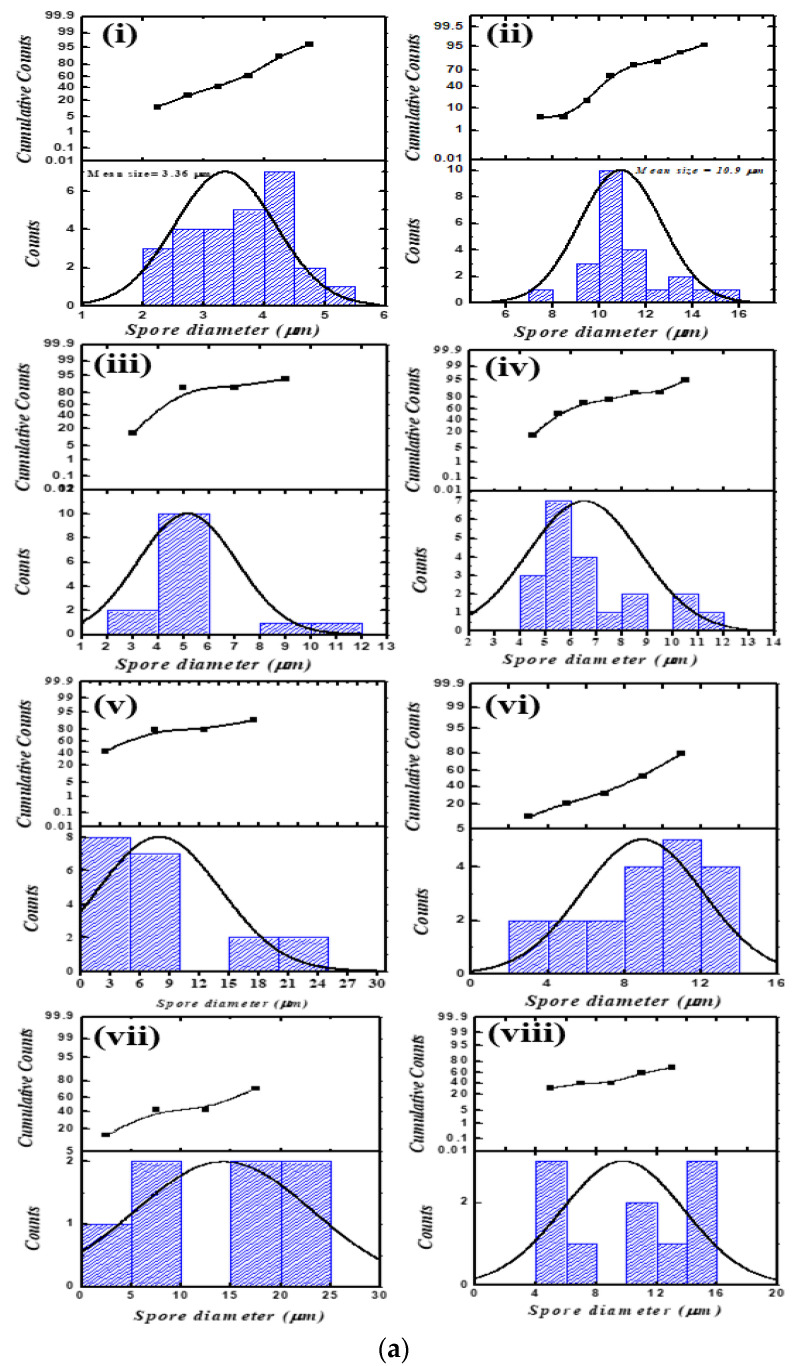

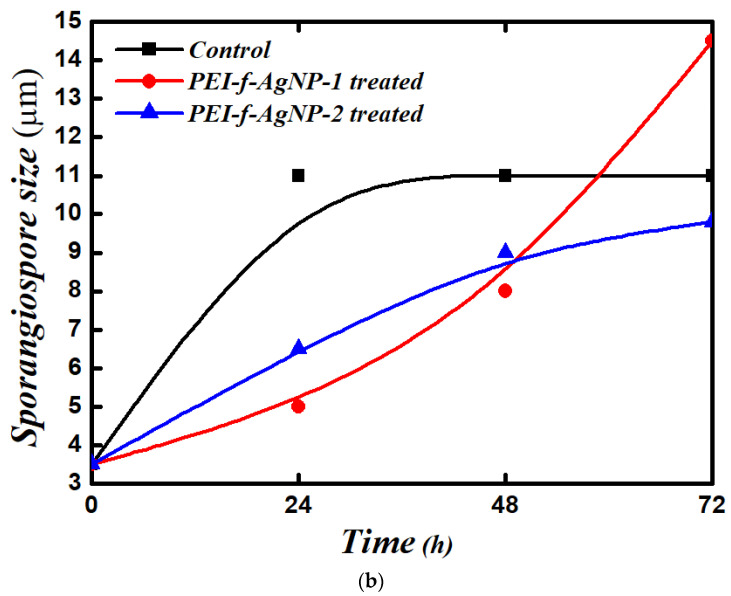
(**a**) Size distribution histogram of PEI-f-Ag-NPs treated sporangiospores: (**i**) freshly harvested sporangiospores, (**ii**) fully germinated sporangiospores at 24 h, (**iii**) PEI-f-AgNP-1 treated sporangiospores at 24 h, (**iv**) PEI-f-AgNP-2 treated sporangiospores at 24 h, (**v**) PEI-f-AgNP-1 treated sporangiospores at 48 h, (**vi**) PEI-f-AgNP-2 treated sporangiospores at 48 h, (**vii**) PEI-f-AgNP-1 treated sporangiospores at 72 h, and (**viii**) PEI-f-AgNP-2 treated sporangiospores at 72 h. (**b**) Average sporangiospore size during germination at various times (24–72 h) in PEI-f-Ag-NPs treated and control sporangiospores.

Another interesting morphological change was observed; a small population in PEI-f-Ag-NP 1 treated sporangiospores became swollen continuously with time as compared to their untreated controls without forming germ tubes (Figure 4a(i–viii)). The remaining larger populations exhibited similar properties in respect to size, corresponding to control sporangiospores (untreated) without germ tube formation, and were considered dead after 72 h of incubation. The increase in sporangiospore average diameter in PEI-f-Ag-NP 1 and 2 treated sporangiospores along with control over time is indicated in Figure 4b. Generally, the ungerminated sporangiospores were 3–5 μm in size. They absorbed water from the surrounding medium and swelled over time and reached 10 µm in size [23,24].

**Figure 5:**
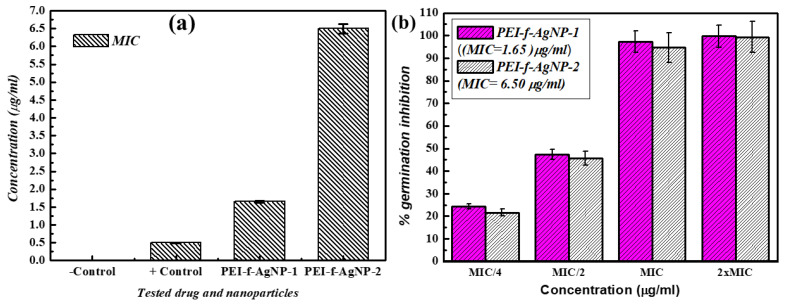
Minimum inhibitory concentration of PEI-f-Ag-NPs against sporangiospores of *R. arrhizus.* (**a**) and (**b**) representing the % sporangiospores germination inhibition by PEI-f-Ag-NPs in a dose-dependent manner (PEI-f-AgNP-1 MIC/4 = 0.41, MIC/2 = 0.82, MIC = 1.65, and 2 × MIC = 3.30 μg/mL) and PEI-f-AgNP-2 (PEI-f-AgNP-2 MIC/4 = 1.62, MIC/2 = 3.25, MIC = 6.50, and 2 × MIC = 13.0 μg/mL). Data are presented as per cent error bar.

The MIC values were determined by a two-fold serial dilution method. Both PEI-f-Ag-NPs (1 and 2) exhibited anti-sporangiospore activity with different killing dynamics. It was observed that, despite being larger in particle size, PEI-f-AgNP-1 had a much lower MIC value (1.65 µg/mL) than PEI-f-AgNP-2 (6.50 µg/mL) (Figure 5a).

### 3.3. Sporangiospore Germination Attenuation Studies

The sporangiospores of *R. arrhizus* germinate and develop into a mature vegetative body, which further forms sporangiospores. These dispersed sporangiospores can be found in air, water, and soil, causing infection after inhalation or ingestion by an immunologically compromised person. The germination inhibition of sporangiospores is important to prevent infection. This study evaluated the *R. arrhizus* sporangiospore germination attenuation by PEI-f-Ag-NPs. Due to the differences in MIC values of PEI-f-AgNP-1 and 2, a variable concentration was taken as MIC/4, MIC/2, 1xMIC, and 2xMIC (PEI-f-AgNP-1: 0.41, 0.82, 1.65, and 3.30 µg/mL, PEI-f-AgNP-2: 1.62, 3.25, 6.50, and 13.0 µg/mL, respectively) for 72 h; the % germination inhibition efficiency was determined. The results showed that PEI-f-Ag-NPs displayed dose-dependent germination attenuation effects on sporangiospores, as shown in Figure 5b. The PEI-f-AgNP-1 treated sporangiospores germination inhibition rates were 24.33%, 47.2%, 96.85%, and 99.9% for MIC/4, MIC/2, 1 × MIC, and 2 × MIC doses, respectively, at 24 h; however, no increment in germination rate was observed at later time points (e.g., 48 and 72) (Figure 5b). Similarly, PEI-f-AgNP-2 treated sporangiospores showed the germination inhibition at 21.66%, 45%, 94.33%, and 99%, respectively, for MIC/4, MIC/2, 1 × MIC, and 2 × MIC doses at 24 h of incubation (Figure 5b).

### 3.4. Confocal Lesser Scanning Microscopy (CLSM) Studies

**Figure 6:**
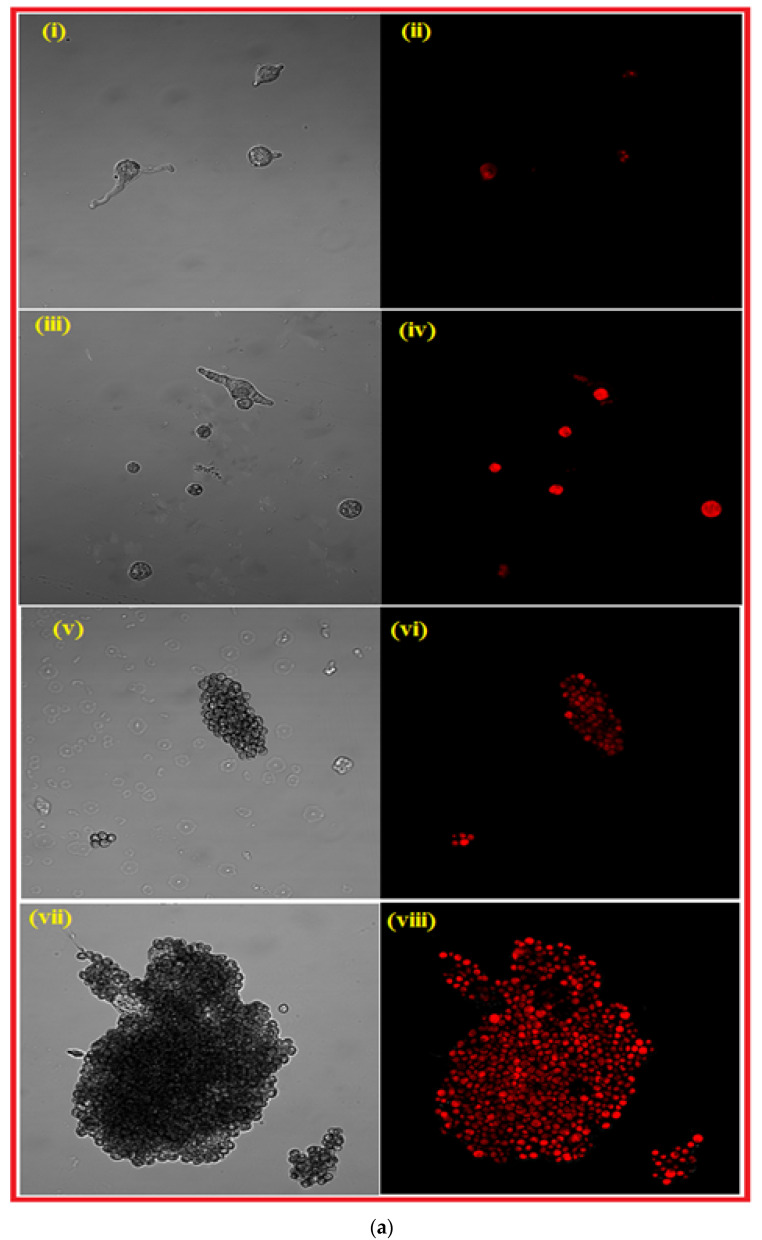

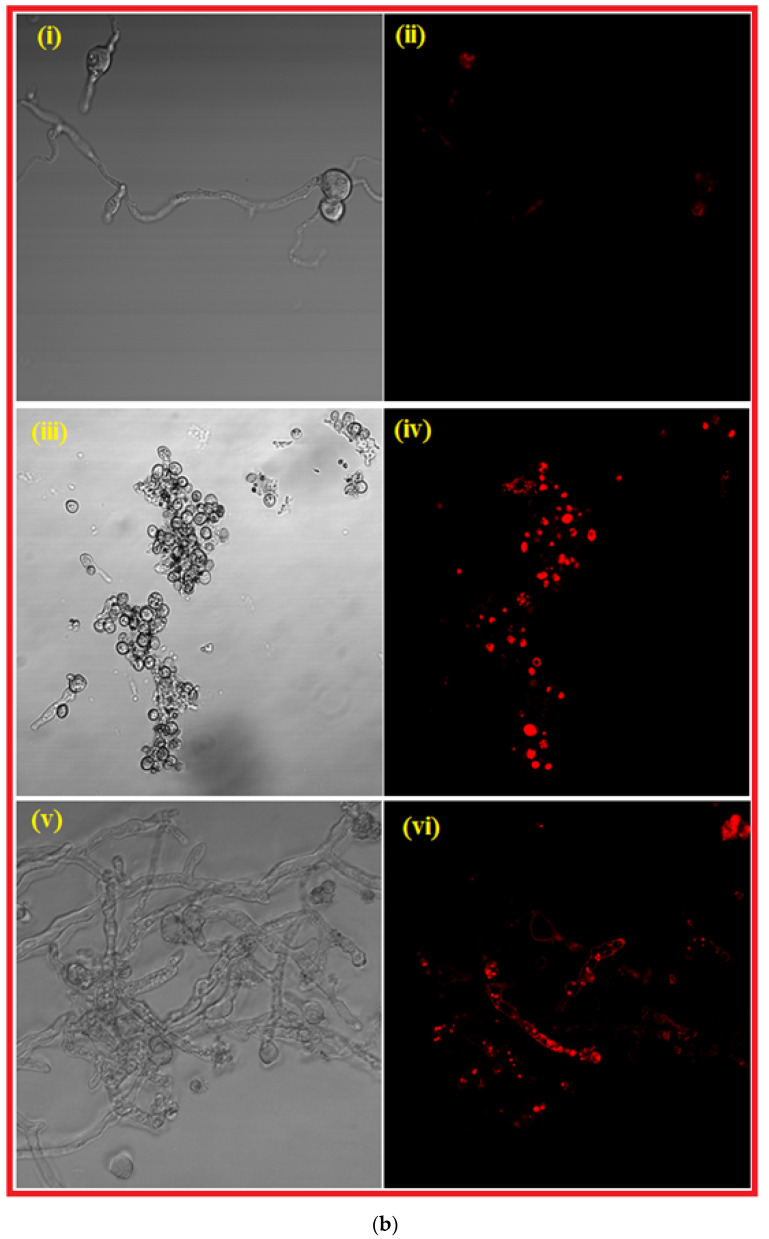

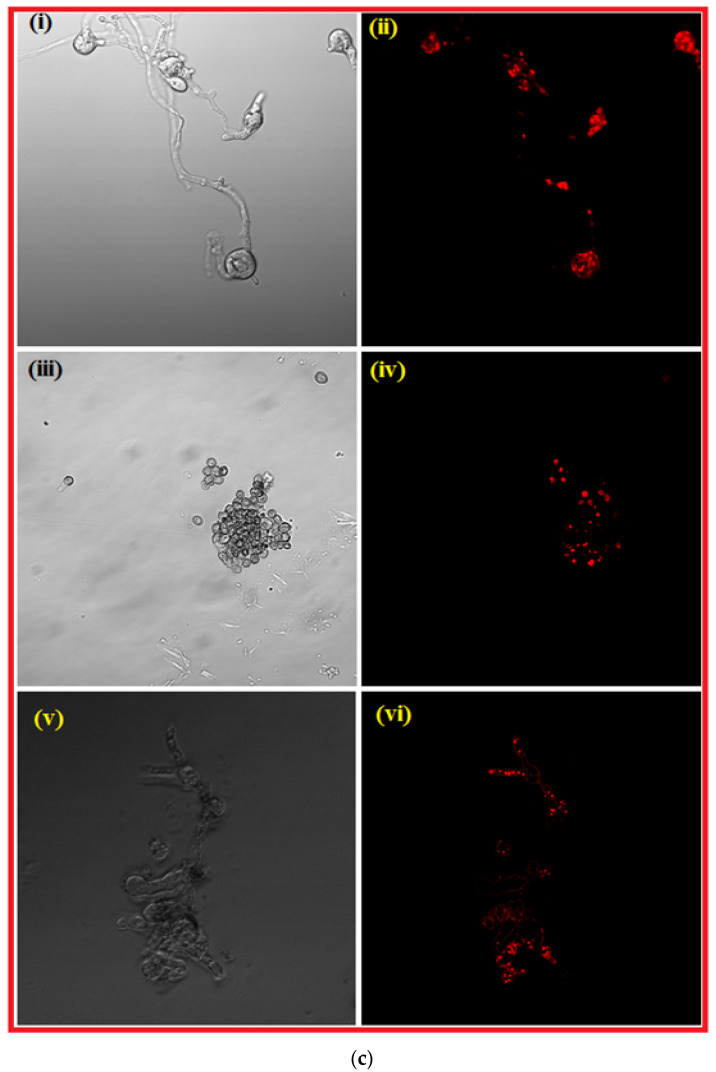
(**a**). Showing the confocal laser scanning microscopy of *R. arrhizus* sporangiospores treated with PEI-f-Ag-NPs and control stained with PI dye, for 24 h along with their respective DIC images. (**i**,**ii**) control sporangiospores; (**iii**,**iv**) treated with amphotericin B; (**v**,**vi**) treated with PEI-f-AgNP-1 and (**vii**,**viii**) treated with PEI-f-AgNP-2. (**b**). Showing the confocal laser scanning microscopy of *R. arrhizus* sporangiospores treated with PEI-f-Ag-NPs and control stained with PI dye, for 48 h along with their respective DIC images. control sporangiospores (**i**,**ii**); (**iii**,**iv**) treated with PEI-f-AgNP-1 and (**v**,**vi**) treated with PEI-f-AgNP-2. (**c**). Showing the confocal laser scanning microscopy of *R. arrhizus* sporangiospores treated with PEI-f-Ag-NPs and control stained with PI dye, for 72 h along with their respective DIC images. (**i**,**ii**) control sporangiospores; (**iii**,**iv**) treated sporangiospores with PEI-f-AgNP-1; and (**v**,**vi**) treated with PEI-f-AgNP-2.

The anti-sporangiospores activity of PEI-f-Ag-NPs against mucormycotic agent *R. arrhizus* sporangiospores was further confirmed using fluorescent dye PI. The PI dye is membrane-impermeable and commonly used to stain cells with damaged or compromised membranes; red fluorescence emission usually indicates dead cells [18]. The PEI-f-Ag-NP 1 and 2 treated sporangiospores were incubated at their respective MIC values for 72 h. In addition, an aliquot was taken at different time intervals (24, 48, and 72 h) and stained with PI along with the control; it was observed using a confocal laser scanning microscope. The results showed that most of the sporangiospores were nonviable (PI-stained) within 24 h of treatment, as shown in Figure 6. At 48 and 72 h of treatment, germinated but nonviable mycelia were observed (Figure 6b,c).

### 3.5. Ultrastructural Investigation of Sporangiospores by SEM

**Figure 7:**
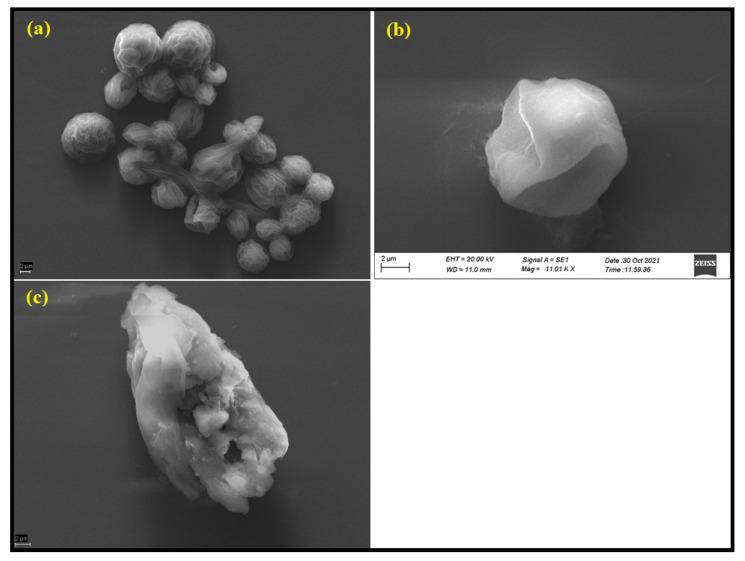
Scanning electron microscopy images of sporangiospores: (**a**) control, (**b**) treated with PEI-f-AgNP-1, and (**c**) treated with PEI-f-AgNP-2 for 24 h.

The above results imply that the incubation of *R. arrhizus* sporangiospores with PEI-f-Ag-NPs is essential for the inactivation of sporangiospores. Thus, scanning electron microscopy (SEM) was used to explore the interaction between various surface charges and particle sizes of PEI-f-Ag-NPs and sporangiospores at their respective MIC values at 24 h of incubation as shown in Figure 7. The results showed that sporangiospores treated with water (control) possessed natural, intact, and characteristic longitudinal ridges as well as an unbroken cytoarchitecture (Figure 7a). However, PEI-f-Ag-NP-1 treated at its MIC (Figure 7b) showed the complete disappearance of longitudinal ridges, shrinkage, and damaged surface architecture. PEI-f-Ag-NP-2 treated sporangiospores were completely fractured, deformed, and collapsed as shown in Figure 7c.

**Figure 8:**
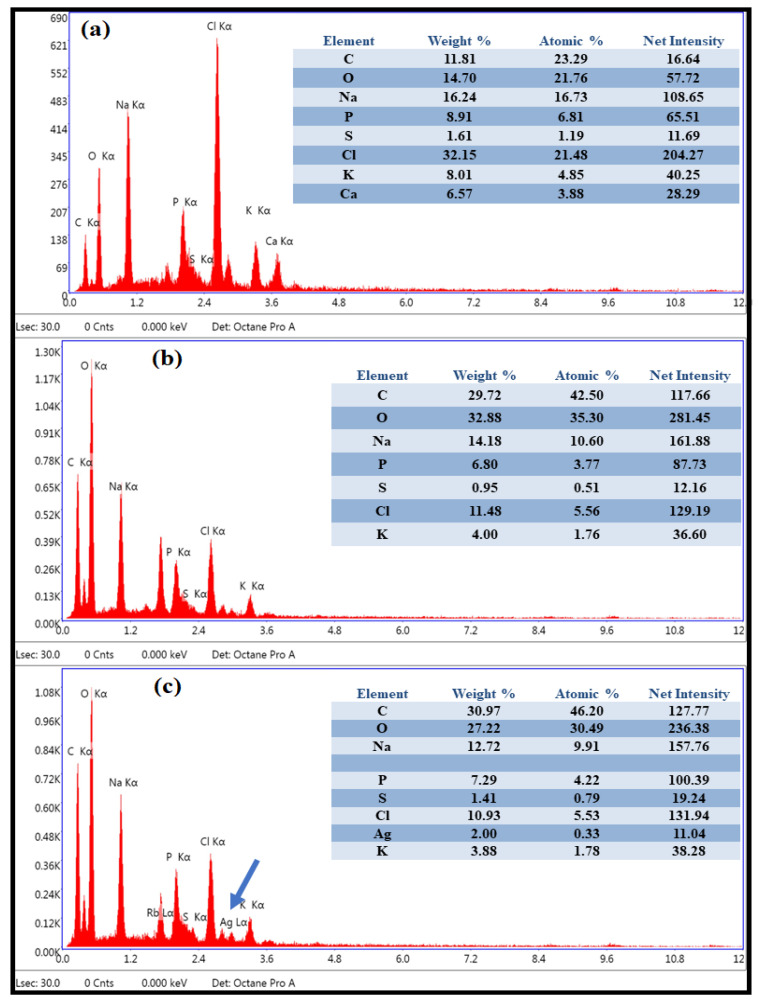
Elemental analysis (EDS) of sporangiospores: (**a**) control, (**b**) treated with PEI-f-AgNP-1, and (**c**) treated with PEI-f-AgNP-2 for 24 h.

Further, the EDS analysis of PEI-f-Ag-NP 1 and 2 treated sporangiospores was used to assess the presence of elemental silver distribution in the sporangiospores. In PEI-f-AgNP-1- and water-treated sporangiospores, no elemental silver was detected, as shown in Figure 8a,b. In contrast, elemental silver was detected in PEI-f-AgNP-2-treated sporangiospores (Figure 8c), suggesting different interaction dynamics of each type of PEI-f-Ag-NPs.

### 3.6. SERS Studies of PEI-f-Ag-NPs Interaction with Sporangiospores

**Figure 9:**
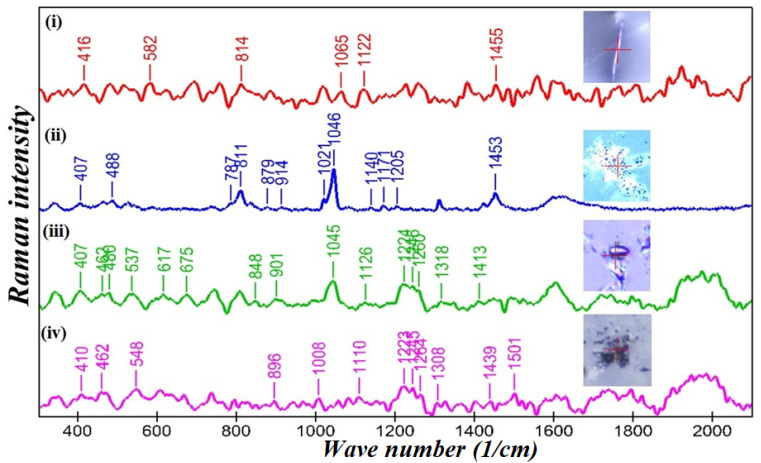
Comparison of the surface enhanced Raman spectra of *Rhizopus arrhizus* sporangiospores treated with PEI-f-Ag-NPs for 24 h. (**i**) Raman spectrum of mycelium, (**ii**) SERS spectrum of freshly harvested sporangiospores, (**iii**) SERS spectrum of sporangiospores incubated with PEI-f-AgNP-1, and (**iv**) SERS spectrum of sporangiospores incubated with PEI-AgNP-2 with corresponding confocal Raman microscopic photographs (inset). Band peaks are assigned for matched biomolecules.

A vibrational spectroscopy method, such as Raman spectrometry, provides complex system fingerprint characteristics for various chemical and biochemical components [25,26]. The Raman signal is relatively weak, limiting its applicability. Surface-enhanced Raman spectroscopy possesses extremely high sensitivity and may be used to obtain precise information on fingerprint molecules. The sensitivity level can be enhanced 10^3^–10^6^ fold compared with the normal Raman scattering process [25,26]. Changes in the chemical composition of lipids, proteins, nucleic acids, and cellular metabolites can be monitored using SERS [27,28,29,30]. It should be noted that previous Raman spectroscopy studies of Ag-NPs and fungi were principally performed to detect isolated fungi and distinguish different fungal species via SERS. The present work, we believe, is the first report using Raman spectroscopy to understand the in-situ interaction of PEI-f-Ag-NPs with *R. arrhizus* sporangiospores. In this study, the SERS was performed on sporangiospores incubated with PEI-f-AgNP-1 and 2 for 24 h, as shown in Figure 9. Similarly, the germinated and ungerminated sporangiospores were examined as control samples. Several biomolecule Raman shifts were observed at various wavenumbers in PEI-f-AgNP-1 and 2 treated sporangiospores, as compared to freshly harvested ungerminated and germinated sporangiospores at 24 h. A list of biomolecules observed at a particular wavenumber in germinated mycelia, ungerminated sporangiospores, and PEI-f-Ag-NPs treated samples is provided in Supplementary Table S1. Matched specific biomolecules at particular wavenumbers, such as amino acids (L-proline and L-alanine in PEI-f-AgNP-1-treated samples and L-alanine and phenylalanine in PEI-f-AgNP-2-treated samples), nucleotides, fatty acids, and sugar complexes, suggested sporangiospore damage. The SERS results showed the presence of trehalose, D-galactosamine, L-alanine, Acetyl CoA, and amylopectin molecules at 407, 462, 480, 1243, and 1264 cm^−1^ in PEI-f-AgNP-1 and 2 treated samples (Figure 9(iii,iv)). However, the trehalose molecule was also identified in freshly harvested sporangiospores and germinated mycelial control at 407 and 1455 cm^−1^ (Figure 9(i,ii)). This type of comparison requires additional studies, which are currently under way.

## 4. Discussion

Mucormycosis is a life-threatening infection caused by *R. arrhizus*, a saprophytic fungus, which commonly occurs in diabetic and hematopoietic stem cell transplant patients. During the second wave of COVID-19 (March–June 2021) in India, ~51,775 mucormycosis cases were reported, for which uncontrolled hyperglycemia was considered the most common predisposing risk factor. The free-floating environmental sporangiospores enter the human body through inhalation, where they primarily colonize and infect the paranasal sinuses, nasal cavity, and orbit. In this study, we have investigated the anti-sporangiospore activity and impact of polyethyleneimine functionalized silver nanoparticles on the germination of sporangiospores and their antifungal mechanism.

Polyethyleneimine is a polycationic, hydrophilic polymer, which is used for DNA transfection of mammalian cells, drug delivery, and material coatings in the biomedical and industrial fields. We have documented the synthesis of polyethyleneimine functionalized silver nanoparticles in previous studies [17]. Apart from polyethyleneimine, other biocompatible silica-containing organic polymers, such as organotrialkoxysilane (e.g., 3-aminopropyltrimethoxysilane and 3-glycidoxytrimethoxysilane), along with organic reducing agents cyclohexanone and formaldehyde, are being used for the controlled synthesis of monometallic, bimetallic, and trimetallic noble metal nanoparticles [22]. During the synthesis of NPs, we used methanol and an organic monomer, 1-vinyl 2 pyrrolinone, to reduce the silver cation reactivity with PEI and protect the synthesized nanoparticles from agglomeration. Subsequently, synthesized PEI-f-Ag-NPs were reconstituted in ultra-pure HPLC-grade water to determine their anti-sporangiospore activity.

The synthesized PEI-f-Ag-NPs showed UV-Vis absorption maxima at 420 and 408 nm, respectively (Figure 1A). The molecular weight of polyethyleneimine affects the size of silver nanoparticles, which resulted in this shifting; the size was confirmed by TEM (Figure 1B). It was interesting to observe that the higher molecular weight PEI (*Mw* 700,000 Da) led to the synthesis of larger-sized PEI-f-AgNP-1 (~20.4 ± 5.9 nm); in comparison, the PEI-f-AgNP-2 synthesized with lower molecular weight PEI (65,000 Da) had a size of ~4.2 ± 0.7 nm. Apart from the molecular weight variation, the PEI concentration also controls the size of synthesized NPs; this relationship was evident from UV-Vis spectrophotometry (as reported previously) [17,31]. The molecular weight of PEI depends on the number (n) of monomers, which, proportionately, increases the net cationic charge of the molecule. The ζ value of both PEI-f-Ag-NPs was positive because of the greater number of amine groups exposed on the PEI surface [31]. Therefore, the ζ potential of synthesized PEI-f-Ag-NPs depends on the molecular weight of PEI (Figure 2). However, Ortega et al. reported on the mechanism of PEI functionalized silver nanoparticles in detail and suggested that the PEI-mediated redox reaction promotes nucleation and growth of NPs, resulting in the formation of size-dependent particles [31]. Initially, the silver cation (Ag^+^) forms a complex with non-bonding electrons (nitrogen atoms) of the PEI amine group. Such bonding leads to the nucleation and growth of NPs. To promote the complete redox reaction, a microwave oven was used to yield stable NPs. However, the role of molecular weight of PEI in the size determination of NPs has not been fully elucidated and requires a more detailed investigation.

We used a clinical strain of *R. arrhizus*, which was isolated from a patient suffering from COVID-19-associated mucormycosis, to explore sporangiospore survival and germination behavior in the presence of the synthesized PEI-f-Ag-NPs. Both PEI-f-AgNP-1 and 2 showed significant anti-sporangiospore activity and attenuated germination within 24 h of exposure. During incubation of sporangiospores with PEI-f-AgNP-1, we observed an interesting behavior in which a few populations of sporangiospores were swollen (~14 μm in size) much larger as compared to the control with increasing time. Usually, the swollen sporangiospores result from water absorption before germ tube formation [23,24]. PEI-f-AgNP-1 exposure resulted in the killing of most of the sporangiospores; a few remained alive, which failed to germinate, despite having altered size and shape (Figure 3 and Figure 4a,b).

In contrast, PEI-f-AgNP-2-treated sporangiospores did not show any abnormal behavior as most of the cells were fractured. This difference in the anti-sporangiospore activity of the PEI-f-Ag-NPs is due to differences in the cationic surface charge as a function of the molecular weight of PEI. Although the results followed the MIC of both PEI-f-Ag-NPs, PEI-f-AgNP-1 had a lower MIC (1.65 μg/mL) than PEI-f-AgNP-2 (6.5 μg/mL). Despite having a higher MIC, PEI-f-AgNP-2 showed a significant detrimental impact on the cytoarchitecture of *R. arrhizus* sporangiospores. This finding can be explained by the difference in the charge and size of the PEI-f-Ag-NPs. PEI-f-AgNP-1 is larger in size and highly cationic (~20.4 nm ± 5.9, charge +25 mV). It usually fails to enter the sporangiospores (compared to PEI-f-AgNP-2); however, this large size NPs probably undergoes frequent collisions and forms strong electrostatic interaction with sporangiospores (Figure 5a).

Further, the anti-sporangiospore activity of both PEI-f-Ag-NPs was confirmed by a confocal laser scanning microscopy study that involved staining of the sporangiospores with PI dye. The PI fluorophore binds with cellular DNA and fluoresces red when illuminated at a specific wavelength of light (488/617 nm). The PI molecule cannot cross the intact cell membrane; however, an altered cell membrane allows for cytoplasmic entry. The CLSM study confirmed cell membrane damage within 24 h of incubation with PEI-f-Ag-NPs (Figure 6a). After incubation (e.g., at 48 and 72 h), DNA compaction and fragmentation were observed more commonly in PEI-f-AgNP-2-treated sporangiospores, indicating the internalization of PEI-f-AgNP-2 (Figure 6b,c). It has been reported that highly charged PEI molecules interact more strongly with the cell surface than less cationic charged molecules since a tight electrostatic interaction forms between negatively charged cell surfaces.

Interestingly, the PEI-f-AgNP-2 had a small and low cationic surface charge; it likely entered the cells and caused nucleic acid damage. On the other hand, reactive oxygen species (ROS) generated by PEI-f-AgNP-2 resulted in an alteration of cell membrane permeability with subsequent nucleic acid damage. However, PEI-f-AgNP-1 altered the cell membrane permeability due to strong electrostatic interaction-induced ROS generation. Further, we observed that sporangiospores not exposed to PEI-f-Ag-NPs also had PI staining after 24 h of incubation. This phenomenon is considered an effect of increased water absorption, resulting in increased cell membrane permeability to the dye [23].

Further, to observe the ultrastructural changes in PEI-f-Ag-NPs treated sporangiospores, SEM-EDS was performed (Figure 7). We observed that PEI-f-AgNP-1-treated sporangiospores had a smooth surface without characteristic longitudinal ridges. This finding is probably due to the collapse of membrane potential with subsequent cytoplasmic substance leakage because of strong electrostatic interaction-mediated ROS generation, which further indicates the lack of cellular internalization of PEI-f-AgNP-1 (Figure 7b). In contrast, the smaller PEI-f-AgNP-2 with low cationic surface charge showed cell fracture as observed in SEM-EDS, which supported the light microscopy and CLSM imaging observations. Cell fracture may be attributed to indirect internalization of PEI-f-AgNP-2 via proton channels or porins or direct penetration of the cell wall and membrane, inducing the ROS generation and cytoarchitectural damage (Figure 7c). Similar observations have been confirmed by SEM-EDS analysis of PEI-f-Ag-NPs-treated samples (Figure 8a–c). Furthermore, SEM-EDS analysis has shown the absence of elemental silver in PEI-f-AgNP-1-treated sporangiospores, which may be due to the washing out of nanoparticles from the sporangiospore surface during sample preparation. In contrast, sporangiospores treated with PEI-f-AgNP-2 contained elemental silver, further indicating the internalization of PEI-f-AgNP-2 nanoparticles within the sporangiospores.

Raman spectroscopy is a non-destructive, rapid technique requiring only small sample volumes, which is associated with nearly no water interference. The bands in Raman spectra are well resolved compared to similar spectroscopic techniques- such as infrared spectroscopy [30]. De Gussem et al. analyzed *Lactarius* spore composition, and Edwards et al. studied lichens using Raman spectroscopy methods [32,33]. To understand the interaction of PEI-f-Ag-NPs with sporangiospores, a SERS study was performed on PEI-f-Ag-NPs-treated sporangiospores and germinated mycelium; freshly harvested sporangiospores served as a control (Figure 9). The results confirmed cell wall/membrane damage and cytoplasmic leakage by PEI-f-Ag-NPs. Among the fingerprint biomolecules, few common metabolites and nucleotides were identified in the treated sporangiospores (e.g., trehalose (407 cm^−1^), cytosine (537, 582 cm^−1^), thymine (617 cm^−1^), ꞵ-carotene (1008 cm^−1^), acetyl CoA (1243 cm^−1^), and amylopectin (1264 cm^−1^)). The presence of glutathione and trehalose in PEI-f-AgNP-1-treated sporangiospores indicated nanoparticle-induced ROS generation [34,35]. Trehalose is a nonreducing sugar-containing two glucose subunits with an α, α-1,1-glycosidic linkage [36]. In fungi, trehalose is present in all structures of the life cycle, such as spores, fruiting bodies, vegetative cells, and hyphae, which rapidly deplete after germination. In addition, trehalose serves a role in central fungal metabolism and in responses to specific environmental stress. Trehalose forms hydrogen bonds with surrounding water molecules and functions as a replacement for water by interacting with phospholipids or other macromolecules in the cell membrane under stress conditions [36]. This study identified trehalose in freshly harvested and PEI-f-Ag-NPs-treated sporangiospores at 407 cm^−1^. However, it was also present in the germinated mycelial control at 1455 cm^−1^, which indicated molecular changes, such as bond stretching or bending (Figure 9). The identification of cellular metabolites, such as acetyl coenzyme A and ꞵ- carotene, indicated the presence of a damaged cell wall/membrane in treated sporangiospores. In *Mucorales*, ꞵ- carotene synthesizes sporopollenin polymers, protecting sporangiospores in a stressful environment. N-acetylglucosamine, detected in PEI-f-AgNP-1-treated sporangiospores at 1126 cm^−1^, is the cell wall component of bacteria and fungi; it was reported previously in yeast cells via SERS spectroscopy at 1125 cm^−1^ [34]. Thus, shifts in the Raman spectra of N-acetylglucosamine have been assigned to stretching in CO and CC bonds and out-of-plane bending in CH bonds, suggesting sporangiospores wall damage. Further, a D-galactosamine peak was observed in both PEI-f-Ag-NPs-treated sporangiospores at 462 cm^−1^; however, it was absent in the mycelial and sporangiospore control samples, indicating surface structural deformations. In addition, we also observed considerable biomolecular differences between freshly harvested sporangiospores and fully germinated sporangiospores mycelia. Very few biomolecules identified in germinated mycelia (e.g., glycerol (416 cm^−1^), succinic acid (582 cm^−1^), 14 methylpentadecanoic acid (814 cm^−1^), and triolein (1065 cm^−1^)). The Raman signal in treated and control samples was somewhat low and needed more process optimization for the analysis of molecular changes, such as bond stretching and bending in molecules. However, a detailed study is under way.

### Possible Anti-Sporangiospores Mechanism of PEI-f-Ag-NPs

**Figure 10:**
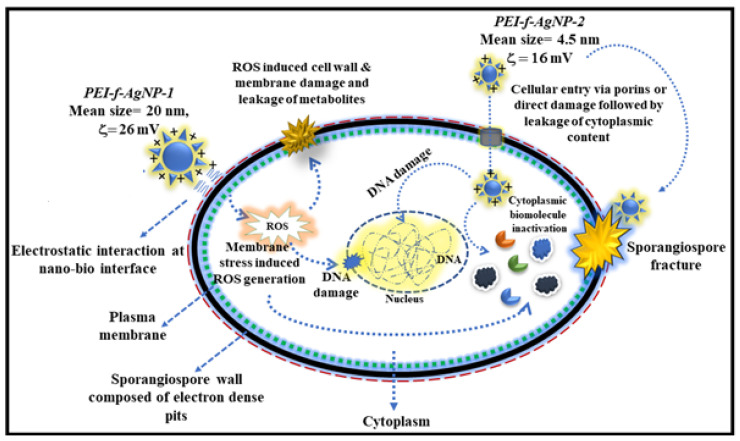
Diagrammatic representation of the possible mechanism of anti-sporangiospore activity of PEI-functionalized silver nanoparticles.

Our previous study reported that PEI functionalized silver nanoparticles as a function of their molecular weight interacted with the surface-expressed proteins of *Acinetobacter bauminnii* and quenched their autofluorescence [17,37]. However, in our present study, the anti-sporangiospore mechanism on *R. arrhizus* indicates a different pathway. The PEI functionalized silver nanoparticles (PEI-f-AgNP-1 and 2) differed in their size and zeta potential, as discussed above, as a function of the molecular weight of PEI. Due to the variable physical properties of PEI-f-Ag-NPs, the mode of action against the sporangiospores may be different. As our findings discussed above, it is plausible that PEI-f-AgNP-1 interacts electrostatically at the nano-bio interface with negatively charged sporangiospores, resulting in stress-induced ROS generation that leads to the inactivation of biomolecules with subsequent swelling and cellular leakage. In contrast to PEI-f-AgNP-1, PEI-f-AgNP-2 had a smaller size and low zeta potential and, as such, may enter the cell directly as a nanoparticle bullet or indirectly through proton channel/porins. The proposed model is shown in Figure 10.

## 5. Conclusions

The synthesis and impact of surface-functionalized silver nanoparticles derived from various molecular weights of polyethyleneimine have been demonstrated against *R. arrhizus* sporangiospores. The functionalized silver nanoparticles showed variable size and surface charge as a function of the PEI molecular weight. Further, functionalized silver nanoparticles (PEI-f-AgNP-1 and 2) showed excellent anti-sporangiospore activity and germination inhibition efficiency. Mechanistically, PEI-f-AgNP-1 exerts its effect on sporangiospores by establishing a strong electrostatic interaction with the negatively charged surface and generating stress, followed by induction of ROS generation, which, ultimately, damages the cell wall/membrane. On the other hand, due to its small size and less cationic nature, PEI-f-AgNP-2 could enter the sporangiospores. The stability and dispersibility of PEI-functionalized silver nanoparticles in aqueous and nonaqueous solvents offer the potential for various biomedical applications, such as indoor air sterilization products, surface sterilization products, biomaterial coatings, and food container coatings, to prevent sporangiospores contamination.

## Data Availability

The datasets generated during and/or analyzed during the current study are available from the corresponding author on reasonable request.

## Supplementary Materials

The following supporting information can be downloaded at: https://www.mdpi.com/article/10.3390/nano12132235/s1, Table S1: List of identified biomolecules in surface-enhanced Raman spectra of *R. arrhizus* sporangiospores treated with PEI-f-AgNP-1 and 2 along with freshly harvested and germinated mycelium controls.
