# Supplementary material for: Size and Zeta Potential Clicked Germination Attenuation and Anti-Sporangiospores Activity of PEI-Functionalized Silver Nanoparticles against COVID-19 Associated Mucorales (Rhizopus arrhizus)
Source: Nanomaterials (Basel). 2022 Jun 29;12(13):2235. doi: 10.3390/nano12132235 (PMC9268377; doi:10.3390/nano12132235)
Supplement: Supplementary file 1 [file nanomaterials-12-02235-s001.zip › nanomaterials-1760456-supplementary.pdf]

nanomaterials

Supplementary Materials

Size and Zeta Potential Clicked Germination Attenuation and Anti-Sporangiospores Activity of PEI-Functionalized Silver Nanoparticles against COVID-19 Associated Mucorales (*Rhizopus arrhizus*)

Atul Kumar Tiwari [1], Munesh Kumar Gupta [2], Govind Pandey [3], Ragini Tilak [2], Roger J. Narayan [4,*] and Prem C. Pandey [1,*]

[1] Department of Chemistry, Indian Institute of Technology (BHU), Varanasi 221005, India; atulkumartiwari.rs.chy19@itbhu.ac.in
[2] Mycology Laboratory, Department of Microbiology, Institute of Medical Sciences, Banaras Hindu University, Varanasi 221005, India; muneshg.micro@bhu.ac.in (M.K.G.); tilakragini28@gmail.com (R.T.)
[3] Department of Paediatrics, King George Medical University, Lucknow 226003, India; gvnd121@gmail.com
[4] Joint Department of Biomedical Engineering, North Carolina State University, Raleigh, NC 27695, USA
* Correspondence: rjnaraya@ncsu.edu (R.J.N.); pcpandey.apc@iitbhu.ac.in (P.C.P.)

Table S1. List of identified biomolecules in surface-enhanced Raman spectra of *R. arrhizus* sporangiospores treated with PEI-f-AgNP-1 and 2 along with freshly harvested and germinated mycelium controls.

Wave Number (cm^{-1})	Mycelial Control	Sporangiospore Control	PEI-AgNP-1 Treated	PEI-AgNP-2 Treated
**407**		Trehalose	Trehalose	Trehalose
**416**	glycerol			
**462**			D-galactosamine	D-galactosamine
**480**			L-alanine	L-alanine
**488**		d- fructose		
**537**			Malic acid, cytosine	
**548**				cytosine
**582**	Succinic acid			
**617**			Thymine	
**675**			Glycerol	
**787**		PEP		
**811**		GSH		
**814**	14 MPDA			
**848**			Stearic acid	
**879**		L- arginine		
**896**				Cellulose
**901**			Amylose	
**914**		β-D-glucose		
**1008**				β carotene
**1021**		Galactosamine		
**1045**			L- proline	
**1046**		L-tryptophan		
**1065**	Triolein			
**1110**				Glycerol

**1122**	L-arginine			
**1126**			N-acetyl glucosamine	
**1140**		Histidine		
**1171**		Acetoacetate		
**1205**		Chitin		
**1223**				Trehalose
**1224**			GSH	
**1243**			Acetyl coA	Acetyl coA
**1264**			Amylopectin	Amylopectin
**1309**				Phenylalanine
**1318**			Glycerol	
**1413**			L-proline	
**1439**				Vaccenic acid
**1453**		L- Proline		
**1455**	Trehalose, Uracil			
**1501**				Methyl palmitic acid
